# Using fish lateral line sensing to improve seismic acquisition and processing

**DOI:** 10.1371/journal.pone.0213847

**Published:** 2019-04-16

**Authors:** Franscisco Wilton de Freitas Silva, Sérgio Luiz Eduardo Ferreira da Silva, Marcos Vinícius Cândido Henriques, Gilberto Corso

**Affiliations:** 1 Programa de Pós-Graduação em Ciência e Engenharia de Petróleo, Universidade Federal do Rio Grande do Norte, Natal, Rio Grande do Norte, Brazil; 2 Programa de Pós-Graduação em Física, Universidade Federal do Rio Grande do Norte, Natal, Rio Grande do Norte, Brazil; 3 Departamento de Ciências Exatas e Tecnologia da Informação, Universidade Federal Rural do Semi-Árido, Angicos, Rio Grande do Norte, Brazil; 4 Departmento de Biofísica e Farmacologia, Universidade Federal do Rio Grande do Norte, Natal, Rio Grande do Norte, Brazil; Universidade Estadual de Maringa, BRAZIL

## Abstract

Bioengineering, which studies the principles and design of biological systems, is a field that has inspired the development of several technologies that are currently in use. In this work, we use concepts from the fish lateral line sensing mechanism and apply them to seismic imaging processing. The lateral line is a sensory system composed of an integrated array of mechanical sensors spanning along the fish body. We compare the array of sensors along body fish with the seismic acquisition, which employs an array of equally spaced identical mechanical sensors to image the Earth’s subsurface. In both situations, the mechanical sensors capture and process mechanical vibrations from the environment to produce useful information. We explore the strategy of using the low-pass and high-pass sensors schema of fish lateral line to improve the seismic technique. We use the full-wave inversion method to compare the conventional acquisition procedure of identical sensors with alternative sets of different sensors, which mimics the fish lateral line. Our results show that the alternate sensors arrangement surpasses the performance of the conventional acquisition method, using just half of the input information. The results point at an image processing technique that is computationally more efficient and economical than the usual seismic processing method.

## Introduction

In general, seismic exploration techniques rely on the collection of massive volumes of data, which requires significant computing power [1]. Recently, a study was conducted on reducing seismic acquisition without losing information by using compressive sensing methods [2]. In this work, we explore ways to reduce data acquisition using fish lateral sensing inspired methods. In fact, the observation and study of animal sensing have inspired scientists and engineers to develop innovative imaging equipment. The most well-known achievements of bioengineering in terms of sensing are the radar and sonar, which were inspired from bats and dolphins sensors [3]. In this paper, we study an animal sensing strategy that improves the imaging methods used in seismology and exploration geophysics.

**Fig 1:**
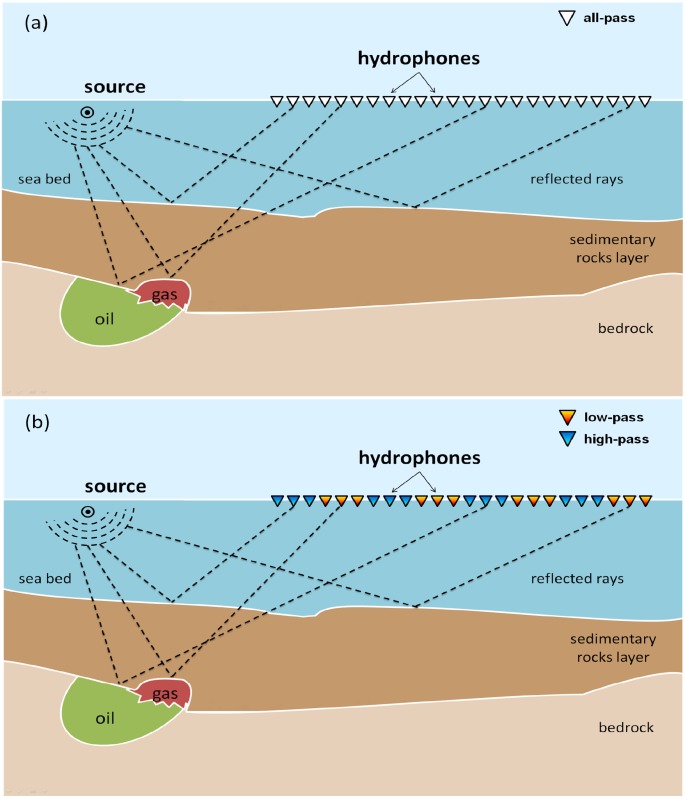
Sketch of a typical seismic exploration showing a source and an array of hydrophones. The propagated waves reflected in the geologic layers are indicated by rays that are reflected at the interface between different media. In (a), we present a conventional schema with identical sensors, while in (b), we show our acquisition design consisting of alternate sets of low-pass and high-pass sensors (represented by triangles of different colors).

The imaging of the subsurface is a topic of great scientific and economical interest [4, 5]. The most important imaging method involves capturing the reflection and refraction of seismic waves artificially produced on the surface. The usual schema consists of an explosive source and several geophones (in aquatic environment, hydrophones are used) that receive the waves reflected from the layers of the Earth’s subsurface. Fig 1(a) illustrates the main design of the seismic exploration methodology showing the wave propagating in the subsurface and being recorded by several hydrophones. In this figure, we depict a set of equally spaced identical sensors, corresponding to the basic acquisition schema in seismic exploration. The use of equally spaced identical sensors is the general schema in geophysics; however, in this work, we question the paradigm of the array of identical sensors.

Full-waveform inversion (FWI) is a seismic imaging methodology that reconstructs the subsurface using the information of the propagated mechanical wave reflected by the geologic layers [6]. The FWI strategy is to solve the wave equation itself and match the result of the computed wave with the observed data from the geophones [5]. The main challenge of FWI is that it is an ill-posed inverse problem. Using the language of inversion problems, many models fit the observed data and we have to choose the best one. Besides, the huge amount of data produced in the geophones and the numerical difficulties of treating large matrices make the FWI a challenging problem.

To tackle the sensor acquisition and FWI problem, we search for inspiration in biology, and study the lateral line, an animal organ sense. This sensing system, which is mostly present in fish but is also found in mollusks and amphibians, is composed of an array of integrated mechanical sensors [7, 8]. The fish lateral line is basically formed of arrays of pressure gradient sensors along the fish body, interconnected by nervous fibers. The fish lateral line has numerous behavioral uses: fish schooling [9], catching preys [10], fish communication [11], orientation to water flows [12], we also call attention to the lateral line imaging or hydrodynamic imaging by the lateral line [13–15]. Moreover, physiological studies show that the mechanical sensors along the lateral line may respond to different frequencies. This fact is rather surprising from a genetic perspective, since it will be cheaper for the organism to design a unique frequency response sensor type; however, actual organisms produce different kinds of pass-band sensors that respond better around 10*Hz*, 40*Hz*, or 100*Hz* [16]. In this manuscript, we explore an array of sensors with distinct frequency response for seismic image processing.

In this work, we develop an innovative method of seismic imaging bio-inspired in the fish lateral line, instead of the trivial all equal sensor line used in exploratory geophysics, we develop an Alternative Frequency Acquisition (AFA) array that responds to different frequency bands. The animal sensing strategy of using regions of low/high frequency band frequency sensors is a consequence of hundreds of millions of years of evolution of animal life. We remark that fishes are among the oldest vertebrate animal taxon, and dates back to 450 million years ago [17]. For the sake of comparison, the early cetacea evolved from terrestrial mammals 50 million years ago, and the dolphins, much later [18]. In this way, the dolphin sonar has a shorter time history than the evolutionary period of the fish lateral line. For this reason, we can hypothesize that nature had plenty of time to adapt an optimal acquisition mechanism for the fish lateral line. Moreover, we understand the sensing acquisition mechanism from a neuroscience perspective, as a mechanism with the ability to acquire, compute, and process information. The animal nervous system has evolved to work as a complex system that has to sense the environment, process the information, and respond to the stimulus quickly and efficiently [19]. Hence, alternate frequency acquisition should be an evolved strategy to maximize the velocity of the computational processing with a minimum of sensory information.

The FWI method in the frequency domain representation shows an ideal framework for implementing the AFA methodology. We remark that our method can be implemented as a seismic processing tool. Instead of performing an alternate high/low frequency acquisition it is possible to implement a high/low frequency filtering as an additional step in the workflow of the seismic processing. Indeed, the improvement of geophysical images during seismic processing is major challenge in geophysical exploration [20, 21]. The rest of the paper is organized as follows: In section 2, we briefly show the FWI theory in both time domain and frequency domain. In addition, we detail our AFA method, which is the core of our work. In Section 3, we present the results, apply the method to a Marmousi image, which is a classical model used in exploration geophysics, and compare it with a conventional approach. In Section 4, we discuss the advantages of our approach and its potential technological implications.

## Methodology

### The full-waveform inversion method

The goal of exploration seismology is to construct an Earth model by collecting and processing data from seismic surveys. The energy for the seismic propagation is provided by an artificial seismic source positioned near the surface, as shown in Fig 1(a). The seismic waves travel through the subsurface, and are reflected by the interfaces between geological layers. Subsequently, they are recorded by a set of receivers near the surface. This process is repeated by repositioning the sources and receivers, thus composing the so-called set of shots. The subsurface imaging is essentially an inverse problem, in which the objective is to construct a model of the physical properties of the medium, such as velocity of propagation and density, from the sparse data provided by the receivers.

An inverse problem that has been gaining considerable attention in geophysics in the last few decades is Full-Waveform Inversion (FWI) [5], which is a technique that can be used to perform high-resolution imaging of the complex subsurface geological structures. The forward problem of FWI consists of imaging the propagation of the seismic wave using the model parameters, which, in this case, are the subsurface wave velocities. On the other hand, the inverse problem consists of finding the model parameters by fitting with the observed seismic data. Tarantola (1984) [22] introduced this method for the first time in the time domain, and the methodology was extended to the Fourier frequency domain [23].

We employ the equation of acoustic waves to represent the wave propagation. For the simplest case of a homogeneous and isotropic medium with velocity *v*(*x*), the wave equation is given by

$$\begin{array}{c} (\nabla^{2}-\frac{1}{v^{2}\left(x\right)}\frac{\partial^{2}}{\partial t^{2}})u\left(x,t\right)=-s\left(x,t\right) \end{array}$$ (1)

where *x* is the position, ∇^2^ is the Laplacian operator, *u* is the pressure field, is the time *t*, and the source term denoted by *s*(*x*, *t*) represents the explosive source. We remark that Eq (1) is a time domain representation of the wave equation. In addition, the physical parameter model consists of *v*(*x*), the acoustic wave velocities along the media.

In this study, we utilize the frequency domain because in this representation, we can naturally adapt the fish lateral line concepts to FWI language. To generate the frequency domain, we apply Fourier time transformation to Eq (1). This frequency representation is also known as Helmholtz equation [24]. The frequency-domain acoustic wave equation is written as

$$\begin{array}{c} (\nabla^{2}+\frac{\omega^{2}}{v^{2}\left(x\right)})\hat{u}\left(x,\omega \right)=-\hat{s}\left(x,\omega \right) \end{array}$$ (2)

where $\hat{u}$ is the pressure field in the frequency domain, *ω* is the angular frequency, and $\hat{s}$ is the Fourier transform of the source term. The operator $\left(\nabla^{2}+\frac{\omega^{2}}{v^{2}\left(x\right)}\right)$ is the impedance matrix [25], or the Helmholtz Operator, which depends on the frequency and the medium velocity.

FWI is formulated as a non-linear optimization problem, which uses the observed **d**_*obs*_ seismic data to find the model parameters (**m**), which, in this case, is the velocity field *v*(*x*) [6]. The relationship between the modeled seismic data **d**_*mod*_ and the model parameters **m** is nonlinear, and is generically denoted by an operator G, defined as

$$\begin{array}{c} d_{mod}=G\left(m\right) \end{array}$$ (3)

The optimization process is conventionally implemented by minimizing the norm of the difference between the observed seismic data (**d**_*obs*_) and the modeled seismic data of the forward problem (**d**_*mod*_(**m**)) for each source-receiver pair. This difference is referred to as the objective function, or misfit function, and it is represented mathematically by

$$\begin{array}{c} \phi \left(m\right)=\sum_{s}^{}\sum_{r}^{}\frac{1}{2}\|d_{\text{obs}}-G\left(m\right)\| \end{array}$$ (4)

where *s* and *r* represent the sources and receivers, respectively. With help of a Fourier transform we go from a time domain representation to a frequency representation. When we work the mathematics of the inverse problem in the frequency representation we operate each frequency in an independent way. Because of that it is natural to play with different set of frequencies during the seismic acquisition and processing. In the frequency representation of the inverse problem, Eq (4) should be calculated independently for each frequency.

To calculate the minimum of the misfit function, we start from a synthetic physical model, the initial model. The optimization process iteratively updates the model parameters by comparing the solution of the forward problem with the observed data. This is performed by multi-scale approach starting from the minimal frequency in Eq (4), and uses the result as the input model for the next frequency inversion in a sequence from the lowest to the highest frequencies to mitigate the non-linearity of the problem [26].

### Alternate Frequency Acquisition (AFA)

A typical seismic survey employs seismic sensors to record the wave energy generated by a seismic source and that reflected by the geological interfaces in the subsurface. In this work, we rather focus on the acquisition frequency settings. The conventional way to perform acquisition is with a set of identical sensors. Inspired by the fish lateral line system, we propose a different way of calculating the objective function in Eq (4), which involves changing the frequency band of operation of the sensors. We called this workflow as the Alternate Frequency Acquisition (AFA) method.

The AFA method is performed using the mathematical operator *G* in Eq (3) during the sampling procedure. We split the set of sensors into two sets; for frequencies lesser than a threshold frequency, *f*^*thre*^, only the first set of receivers is used while for those above the threshold, the other set is employed; the threshold frequency is empirically found. For instance, suppose that we have 12 in-line equally spaced receivers *r*_1_, *r*_2_, *r*_3_…, *r*_11_, *r*_12_. In conventional acquisition, all the receivers record all the frequencies (within its operational limits), i. e., the operation in Eq (3) is performed with all the receivers and the objective function is evaluated for all the receivers. In the AFA-method, only a part of the data is used for the inversion. For instance, in the case of a 3x3 AFA method, we divide the group of 12 receivers into two parts: *r*_1_, *r*_2_, *r*_3_, *r*_4_, *r*_5_, *r*_6_, *r*_7_, *r*_8_, *r*_9_, *r*_10_, *r*_11_, *r*_12_. The underlined receivers only record data with frequencies less than the threshold frequency, 0 < *f* < *f*^*thre*^; these are the low frequency sensors. On the other hand, the not-underlined receivers are the high-pass sensors that record data with frequencies higher than the threshold frequency, *f*^*thre*^ < *f*. A similar arrangement is illustrated in Fig 1(b) with 24 receivers. In this way, in the AFA method, the numerical operation indicated by Eq (3) is performed with only half of the receivers, which substantially decreases the computational cost. As we shall see in the next section, the objective function is evaluated in a very efficient way.

### Numerical implementation

**Fig 2:**
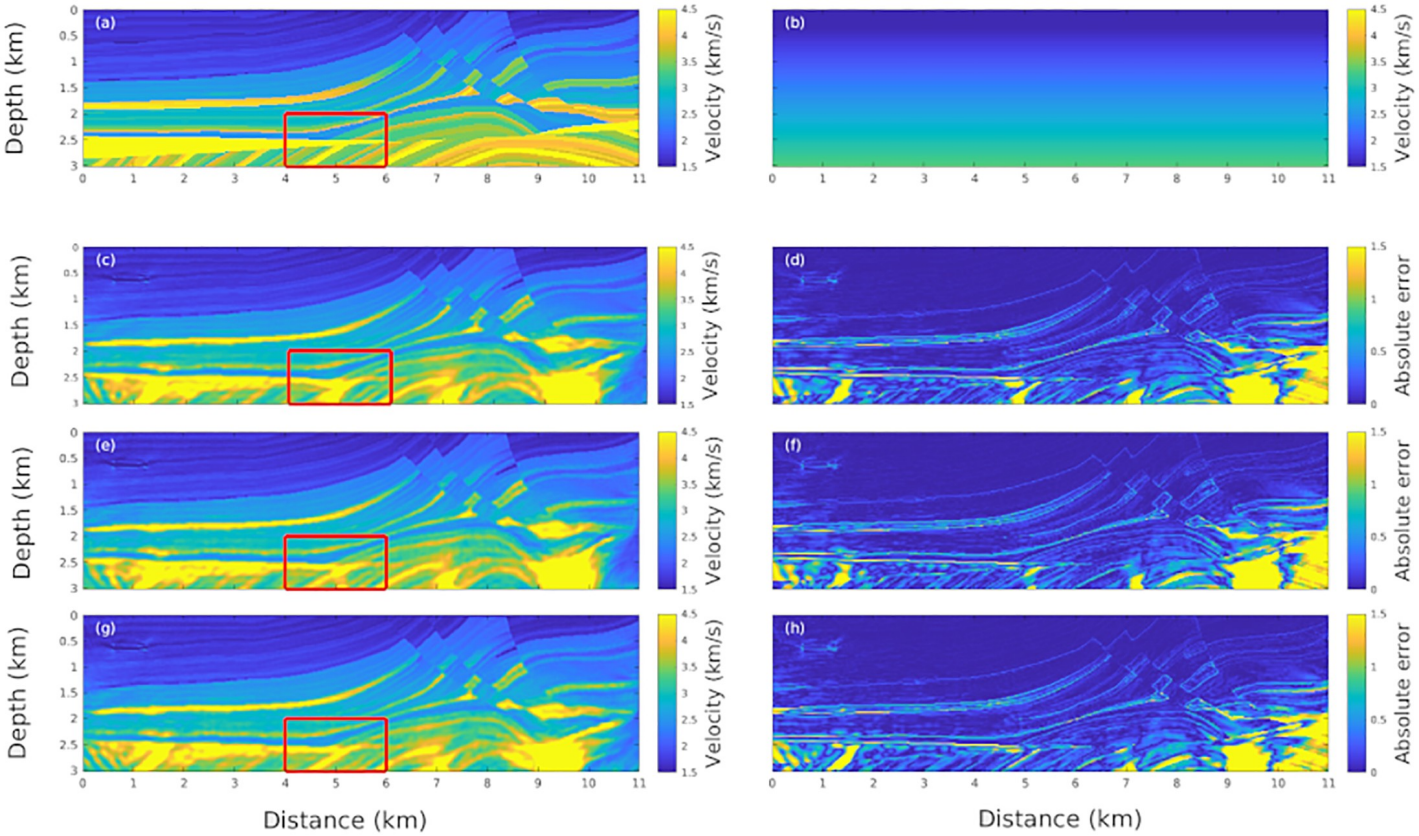
The Marmousi image for different acquisition methods. Panel (a) shows the original Marmousi model. Panel (b) shows the initial input model used in the FWI reconstruction. Panel (c) depicts the Marmousi image constructed using the conventional acquisition procedure while (d) shows the absolute reconstruction error. The reconstruction models using the alternate high/low frequency sensors are shown in panels (e) and (g) for 4x4 and 10x10 arrays; the corresponding absolute errors are shown in (f) and (h).

For the numerical experiments we employed the Marmousi model [27], which is largely used in the literature to test seismic image reconstruction, as shown in Fig 2(a). The Marmousi model contains complex seismic data, and requires advanced processing techniques to obtain a correct subsurface image. In fact, it presents a geometry with many reflectors and strong velocity variations from 1.5 km/s to 4.7 km/s. The initial model used to start the optimization problem is a gradient approximation with velocities from 1.5 to 3.4 km/s, as shown in Fig 2(b).

The Helmholtz operator was discretized [28] on a grid with a spacing of 20 m, using a 5-point finite difference. In addition, we used absorbing boundary conditions [29] to eliminate the border effects caused by undesirable wave reflections. The data was generated using in line fixed acquisition at a depth of 40 m with 55 equally spaced sources located each 200 m from x = 0.1 to 10.9 km. For each source, 68 receivers are located each 160 m from x = 0.1 to 10.82 km at the same sources depth with frequencies from 0.5 to 20 Hz. For frequency inversion, we employed 26 discrete frequencies from 0.5 Hz to 13 Hz. A threshold frequency of 8 Hz was used in all the experiments. To test the methodology, we implemented eleven AFA-configurations from 1x1 to 11x11. In addition, for solving the optimization problem, we used 780 iterations (30 iterations by frequency) and compared the imaging performance with that of the FWI-conventional method. Because of the fast convergence, we adopted the algorithm L-BFGS [30], which is an algorithm of the quasi-Newton method family. All the numerical experiments and figures were performed using Matlab^®^ software.

### Statistical treatment

We study in detail the region of the Marmousi model inside the square box of Fig 2 because it is a challenge to image this structure, in fact, it is a deep region and it is below a salt layer. Moreover, this region is in the center of the image and, as a consequence, it is well illuminated by the acoustic sources. Because it is well illuminated region it should have a good image resolution. We compute the statistics using the model misfit *ψ* = (*m*_*rec*_ − *m*) for *m* (the model values) and *m*_*rec*_ (the reconstructed model estimates). We notice that the function difference *φ* is not the same as *ϕ* presented in Eq (4). The first is defined by the model parameters of the subsurface, while *ϕ* is defined by the observed data from the seismic sensors on the surface. We initially employ three measures to perform the comparison: the arithmetic mean *μ*, the conventional deviation *σ*, and the absolute error *Er* = |*ψ*|. An optimal image reconstruction should have *μ* close to zero, small *σ*, and lower *Er* values. In addition, we show the structural similarity *SSIM*, a measure normally used for computing the similarity between images [31] and the correlation coefficient *r* between the images. A good similarity between images is implied when *r* is close to 1 and the values of *SSIM* are large.

## Results

To test the advantage of using a fish lateral line strategy in exploratory geophysics, we compare different seismic acquisition methods for the same subsurface image. We employ the Marmousi model, Fig 2(a), because it is a well known geophysical image that is largely used in seismic image processing [27]. The initial input model employed in the FWI reconstruction work is plotted in Fig 2(b). For the sake of comparison, we plot the Marmousi image reconstructed using the conventional acquisition method with all equal sensors in panel (c). Panel (d) shows the absolute error *Er* between the original model and the reconstruction. Panels (e) and (g) show the reconstruction models using the AFA method, with alternate high/low frequency sensors in 4x4 and 10x10 arrays. The corresponding *Er* for the last two images are shown in panels (f) and (h).

An overall view of Fig 2 shows that the conventional image reconstruction using all equal sensors is comparable with the AFA image reconstruction, which employs only half of the data. A more detailed view of panels (c) and (e) shows that the AFA method images even better than the conventional reconstruction at the bottom of the image. In fact, a big challenge in exploratory geophysics is deep imaging. With the Marmousi model, it is especially difficult to image below the salt region, which is indicated by a horizontal yellow area in the left part of the figure, situated at a depth of 2.5km. In the square region (horizontal distance from 4km to 5km and depth of 2km to 3km) highlighted in Fig 2(a), the AFA method shows superior performance when compared to the conventional method.

We further analyze the FWI reconstructions with the actual model shown in Table 1. To highlight the differences between the different acquisition procedures, we compute the statistics over a region of the Marmousi model where the differences between the conventional reconstruction and the AFA reconstruction is more accentuated, namely the square region in Fig 2. In Table 1, we perform a comparison between the conventional method and the AFA method. The first point to be considered in this quantitative analysis is that the five quantifiers, namely *μ*, *σ*, *Er*, *SSIM*, and *r* are in good agreement with each other, for instance, low *σ* is associated with low *Er* and large *r*. The second point that deserves attention is that conventional acquisition is worse than any of the AFA methods in the studied region. Finally, we highlight that the best arrangement, for this particular profile is the 4x4 array.

**Table 1:** Table with the main statistics of the numerical experiments. We focus our analysis on the red square region in Fig 2(a). The statistics *μ*, *σ*, and *Er* are based on the misfit between the model and the reconstruction. The quantities *r* and *SSIM* measure similarities between the images. The best results correspond to the 4x4 and 10x10 AFA methods.

*array*	*μ*	*σ*	*Er*(%)	*r*	*SSIM*
conventional	0.640	0.963	22.5	0.556	0.185
1 x 1	0.216	0.484	10.7	0.787	0.458
2 x 2	0.156	0.436	9.21	0.816	0.491
3 x 3	0.189	0.520	10.9	0.751	0.399
4 x 4	0.089	0.391	7.83	0.844	0.558
5 x 5	0.179	0.493	10.5	0.773	0.404
6 x 6	0.210	0.535	11.5	0.745	0.361
7 x 7	0.611	1.184	22.9	0.432	0.124
8 x 8	0.191	0.521	11.1	0.750	0.385
9 x 9	0.145	0.428	8.93	0.821	0.504
10 x 10	0.181	0.489	10.1	0.789	0.437
11 x 11	0.231	0.579	12.4	0.705	0.322

**Fig 3:**
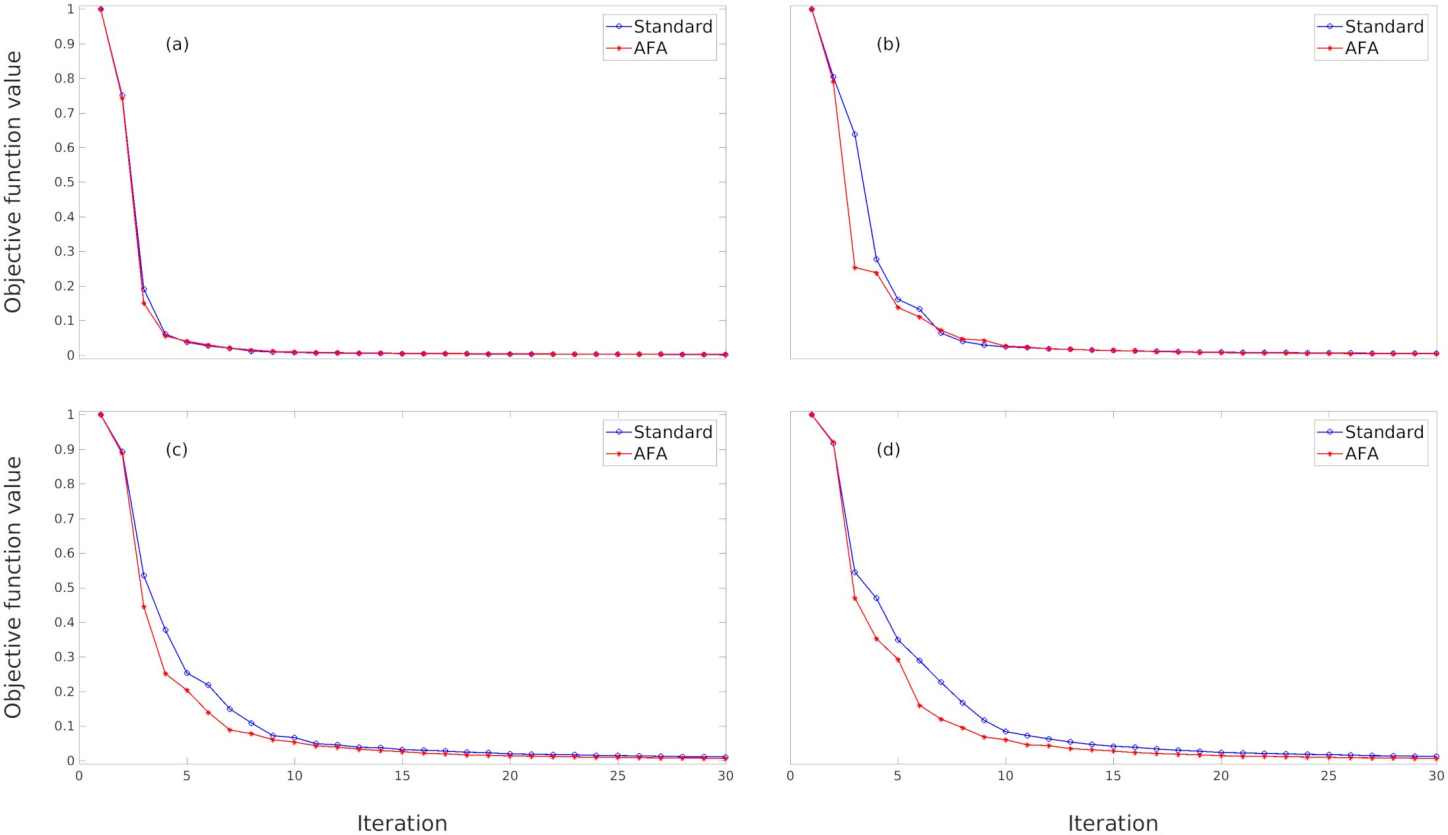
Measuring the convergence of the inverse method: The objective function. We compare the objective function of the conventional acquisition method with that of the AFA 4x4 method, that is, the schema of 4 low-pass and 4 high-pass sensors. We illustrate the convergence for the following frequencies: (a) 3*Hz*, (b) 7*Hz*, (c) 10.5*Hz*, and (d) 13*Hz*.

The performance of the FWI processing algorithm can be visualized using the convergence of the objective function given by Eq (4). A fast and uniform convergence of the objective function is indicative of an efficient computational process during the reconstruction of the inverse problem. In Fig 3, we compare the objective function of the conventional method with that of the AFA method introduced in our work. We use the 4x4 AFA method, which means, an alternate arrangement of 4 low-pass and 4 high-pass sensors in sequence. We chose the 4x4 AFA case because this acquisition arrange shows a good performance according to Table 1. In Fig 3, we have plotted the objective function for several frequencies: (a) 3*Hz*, (b) 7*Hz*, (c) 10.5*Hz*, and (d) 13*Hz*. In all cases, the AFA objective function converges as good or even better than the conventional frequency inversion. Moreover, for high frequencies the AFA method performs significantly better than the conventional method, as shown in Fig 3(c) and 3(d).

## Final remarks

This paper is about an adaptation of fish lateral line sensing acquisition mechanisms to seismic image acquisition and processing. Advances in high-performance computing have allowed the actual development of full-waveform inversion [32], a computation-intensive method that builds seismic images by solving the wave equation. Full-waveform inversion is a methodological background that can used for testing the fish sensing acquisition data in a technological application. Our results using a fish inspired sensing method show that we can satisfactorily reconstruct a seismic image from half as much data as of the conventional method.

Drawing inspiration from bioengineering, we develop a new data acquisition methodology, the Alternate Frequency Acquisition (AFA) method, for frequency FWI seismic imaging. The new method is compared with conventional FWI inversion. The main advantage of the AFA method is a significant reduction in the data needed: instead of using a set of *n* identical sensors, we employ $\frac{n}{2}$ high-pass and $\frac{n}{2}$ low-pass sensors. We tested the seismic inversion for several arrangements of alternating low-pass and high-pass sensors, and the results are quite promising: the reconstructed image is as good or even better than the conventionally acquired one. The superiority of the AFA method is observable in a visual inspection of the seismic image, especially in an image of deep lithofacies. The objective function corresponding to the AFA method converges better than that of the conventional method. Finally, AFA obtains superior overall statistics, average media, conventional deviation, relative error, and structural similarity.

In this paper we introduced the alternative frequency acquisition method. We notice that, instead of playing with the data acquisition by alternating high/low frequency sensors, we could have focused on the seismic processing. In that way, even using the conventional uniform acquisition we could introduce during the seismic processing high/low filter that will produce a similar result to that of the alternating acquisition. We believe that a focus on seismic processing can expand the technological applications of the present paper.

In order to put the seismic imaging in a biophysical context, we compared this sensing technology with echolocation and hydrodynamic imaging. The seismic image process shares many similarities with animal echolocation. In both cases an emitted mechanical wave propagates in the medium, is reflected by reflecting interfaces and the received wave is used to construct an image. In a bat, for instance, the emitted call and the received wave are both processed by spatially closed physiological structures in the head of the animal. In seismic imaging, the emitted wave is typically an explosive source, while the sensors that capture the reflected wave consist of an array of hundreds of elements.

A comparison of seismic imaging with hydrodynamic imaging is also enlightening. Hydrodynamic imaging is an aquatic animal sensing strategy [13] fish swimming in a closed space. An example of this is the blind fish (*Astyanax fasciatus*) in a cave [14, 33, 34] that avoids the walls of the environment during swimming. The spatial dislocation of the fish produces a disturbance in the water pressure along its body. When the fish is close to a wall, or any obstacle, the lines of constant pressure around the fish suffer a disturbance and the fish perceives the obstacles close to its body. The sensing structures of the lateral line are formed by hundreds of mechanical sensors of gradient pressure, the neuromasts. We remark that the hydrodynamic image, as initially described in [13], and even the artificial ones [15, 35, 36], do not sense the water pressure, but rather a gradient of the pressure, and so can detect differences in a pressure field along the body of the organism.

To conclude the comparison between seismic sensing and biophysical mechanisms, echolocation and seismic imaging both construct images using reflected waves, but hydrodynamic imaging does not, it is rather a sensing strategy that perceives disturbances of a field pressure. In this sense, hydrodynamic imaging is closer to electroreception, which perceives changes in the electromagnetic field around the fish’s body. However, the lateral line system, like the seismic image, employs a large set of sensors forming an antenna-like structure to capture mechanical disturbances in the water. The existence of different kinds of neuromasts [7], selective frequency neuromasts along the body [16], or the use of lateral line to matching behavior [11] reveals the great complexity of the lateral line functionalities. The success of using the AFA method in seismic inversion is promising for seismic science and, at the same time, encourages us to persist in understanding the complexity of the lateral line physiology and behavior.
